# Marine and terrestrial biostimulant elicitors of tolerance to cold stress

**DOI:** 10.3389/fpls.2025.1569516

**Published:** 2025-04-08

**Authors:** Eva Regina Oliveira, Aline Nunes, Felipe de Souza Dutra, Gadiel Zilto Azevedo, Alex Ricardo Schneider, Beatriz Rocha dos Santos, Deise Munaro, Sidnei Moura, Giuseppina Pace Pereira Lima, Marcelo Maraschin

**Affiliations:** ^1^ Department of Plant Science, Federal University of Santa Catarina, Florianopolis, Brazil; ^2^ Department of Chemical and Biological Sciences, São Paulo State University, Botucatu, Brazil; ^3^ Department of Biotechnology, University of Caxias do Sul, Caxias do Sul, Brazil

**Keywords:** seaweed, plants, molecules, phytohormones, bioactives

## Abstract

The increasing frequency of adverse environmental events, driven by ongoing climate change, has intensified the search for new technological alternatives in crop production and plant protection. Thermal stress can limit plant adaptation and negatively impact metabolism, physiology, morphology, and yield. Cold stress in plants has been extensively studied and can affect various stages of plant’s life cycle, from seed formation to development, causing damage to cell membranes, impairing cell division, and disrupting water absorption. Consequently, researchers have focused on mitigating the impacts of abiotic stress by investigating bioactive molecules and biostimulants derived from various organisms, which enhance tolerance mechanisms in plants. In aquatic environments, macro- and microalgae have emerged as key sources of plant elicitors, providing extractable molecules such as polysaccharides, polyamines, polyphenols, and amino acids that enhance plant defense responses. Similarly, certain terrestrial plants have shown potential as sources of biostimulant compounds. Thus, this study aims to highlight advancements in crop systems by emphasizing the potential of algae-based and terrestrial biostimulant elicitors in enhancing tolerance to cold stress. Ultimately, the goal is to improve understanding of promising biological models for food production, fostering innovative developments that can contribute to economically and ecologically sustainable technologies.

## 1 Introduction

The search for new bioactive molecules as a solution to reduce agrochemicals in agriculture has led to countless experiments focusing on genetic resources, either from terrestrial or marine environments. In this context, extracts and molecules derived from plants and algae have proven effective as elicitors of plant development and health, enhancing root growth, flowering, and tolerance to adverse environmental conditions (Irani et al., 2021; Ziogas et al., 2022; Riyazuddin et al., 2023).

Bioprospecting approaches are considered sustainable and innovative alternatives to mitigate the harmful effects of climate events on plants of agricultural importance. These approaches range from monitoring methods (Tewari et al., 2017) to interventions in the microbiota, such as the use of bioinoculants developed from organisms native to frozen soils (Pandey and Yarzábal, 2019) and those from desert soils (Zhang and White, 2021).

Particularly in marine environments, microalgae and macroalgae have been identified as potential sources of biofertilizers and biostimulants for plants. They contribute to nutrient transport, plant protection, and growth, besides serving as nutritional additives or elicitors for tolerance induction through bioactive molecules. These include polysaccharides such as ulvans, fucans, alginates, and carrageenans, as well as phytohormones, betaines, polyamines, and amino acids. Compilations by Bello et al. (2021), Poveda and Díez-Méndez (2023), and Di Sario et al. (2025) have highlighted that algal biomass and their constituents have been extensively studied for applications in agriculture (Potijun et al., 2021; Cvetkovska et al., 2022). Algal species from all divisions, including Chlorophyta (green), Rhodophyta (red), and Phaeophyta (brown), have been used for mitigate the impacts of abiotic stress in plants. The results from foliar and soil applications have shown, for instance, increased root emission and shoot growth, along with enhanced defense responses and plant tolerance to (a)biotic stress factors (Sharma et al., 2014; Xu and Leskovar, 2015; Ali et al., 2021; Munaro et al., 2021; Nunes et al., 2024).

In addition to algae, many terrestrial plant species contain bioactive molecules that may benefit cultivated plants. Numerous studies support these findings (Matsumiya and Kubo, 2011; Hayat et al., 2018; Meena et al., 2019; Pacifico et al., 2021; Souza et al., 2021). Plant extracts and oils can serve as additives, biofertilizers, biostimulants, biopesticides, and protectors of crop species (Puglia et al., 2021; Khursheed et al., 2022). The market for these biomolecules has been steadily expanding, driven by farmers’ recognition of their effectiveness in low-carbon food production systems (Rouphael and Colla, 2020; Rakkammal et al., 2023).

Given the potential of algae and plant-based biostimulant compounds, research has focused on developing new technologies to enhance plant tolerance to abiotic stresses, such as water deficit, salinity, and thermal stress – common climatic events that pose significant challenges and lead to major production losses (Bulgari et al., 2019; Deolu-Ajayi et al., 2022; Rakkammal et al., 2023). Among these, cold stress is particularly notable for causing substantial losses, both in the field and post-harvest (Gavelienė et al., 2014; Puzanskiy et al., 2018). However, studies addressing this issue remain limited, especially regarding its impact on global agricultural production (Mahajan and Tuteja, 2005). Thus, this review aims to present and critically discuss the findings of peer-reviewed articles and books that have described and confirmed the effectiveness of seaweed-based and plant biostimulants in mitigating cold stress in various crops.

## 2 Information collection

For the integrative review, several databases were searched, including the Portal de Periódicos Capes, Web of Science, Scopus, and SciELO. This choice was made to ensure a comprehensive collection of information on the use of biostimulants to enhance cold stress tolerance in plants. Additionally, no time filters were applied, however, the selection was limited to peer-reviewed articles or books. This strategy allowed us to expand the search and avoid limitations that could restrict access to relevant information. It is important to note that the specific literature on the use of biostimulants in the context of interest is still relatively scarce, which justifies the need for a broader approach to data collection.

## 3 Biostimulants – definition, market, and legislation

Biostimulants are products used in agriculture that can be applied to plants and soil and are capable of regulating and/or enhancing physiological processes in crops (du Jardin, 2015; Franzoni et al., 2022). Considered environmentally safe and cost-effective alternatives to traditional fertilizers and chemical treatments, they minimize negative impacts on ecosystems while promoting sustainable agricultural practices. They influence plant biochemistry through various metabolic pathways, enhancing morphological yields, increasing quality and shelf life, aiding in the absorption of essential nutrients, and boosting tolerance to abiotic stresses (Ronga et al., 2019).

In industry, the definition of biostimulant was originally proposed in 2012, highlighting that these are plant-based products containing compounds and/or microorganisms that stimulate natural processes when applied to plants and the rhizosphere. Importantly, they do not fit into the regulatory framework for pesticides, as they do not have a direct action against pests (van Oosten et al., 2017). According to du Jardin (2015), a plant biostimulant refers to any substance or microorganism applied to plants to improve nutritional efficiency, enhance tolerance to abiotic stress, and/or improve crop quality characteristics, irrespective of its nutrient content.

Apparently, the term was coined by horticultural experts to describe compounds that promote plant growth without being defined as nutrients, soil amendments, or pesticides (du Jardin, 2015). However, the discussion on this topic can be traced back to 1933, when Professor Filatov in the Soviet Union introduced the concept of biogenic stimulants. Filatov suggested that biological materials from various organisms, including stressed plants, could influence metabolic and energy processes in plant, animal, and even human cells. Other contributions to the discussion came from Blagoveshchensky in 1945, when he described that biogenic stimulants, such as organic acids, would have a stimulating effect due to their dibasic properties, which can increase enzymatic activity in plants. Filatov, however, continued to expand the discussion by not limiting it to organic acids (Yakhin et al., 2017).

In the historical context, Hervé, in 1994, suggested that the development of new “biorational products” should be grounded in chemical, biochemical, and biotechnological principles. These products should be applied in low dosages, be environmentally friendly, and provide reproducible benefits in the cultivation of agricultural species (Yakhin et al., 2017). Supporting this view, Zhang and Schmidt (2000) emphasized the importance of conducting comprehensive and empirical analyses of these products, particularly focusing on hormonal and antioxidant systems. Since then, there have been continuous advancements in the field, encompassing not only the morphological and physiological analyses of treated plants but also the physicochemical characterization of product compositions and the mechanisms of action of biostimulant compounds on the metabolic pathways of crop species (Yakhin et al., 2017).

Building on these foundational insights, recent advancements have highlighted key benefits for crop production, such as enhanced growth, increased yield, and improved fruit quality (Soppelsa et al., 2019; Francesca et al., 2020; Mannino et al., 2020; Navarro-López et al., 2020; Hassan et al., 2021; Gitau et al., 2022; Santini et al., 2022; Riyazuddin et al., 2023). Additionally, there has been progress in mitigating biotic and abiotic stresses (Elansary et al., 2019; Fleming et al., 2019; Campobenedetto et al., 2021; D’Amato and Del Buono, 2021; Desoky et al., 2021; Irani et al., 2021; Riyazuddin et al., 2023; Borella et al., 2023; Kumar et al., 2024), enhancing beneficial soil microbiota and improving nitrogen use efficiency and crop yield (Carillo et al., 2019; Hellequin et al., 2020; Macias-Benitez et al., 2020; Acin-Albiac et al., 2023). Other notable advancements include increased shelf life (Shehata et al., 2017; Mannino et al., 2020; Góes et al., 2021; Ziogas et al., 2022) and the enhancement of bioactive compounds in plants (Rouphael et al., 2018; Jędrszczyk et al., 2018; Mannino et al., 2020; Ashour et al., 2021; Azad et al., 2021; Cirillo et al., 2022; Graziani et al., 2022).

Based on the various scientific evidence already reported, as well as the increased production of organic foods resulting from the growing demand of the population for healthy and sustainable food, biostimulant products have received greater acceptance from the sector, including farmers/producers and government agencies. According to Fortune Business Insights report, the global biostimulant market was valued at USD 2.85 billion in 2021 and it is expected to reach USD 6.69 billion by the end of 2029, exhibiting a compound annual growth rate (CAGR) of 11.43% during the forecast period (Fortune Business Insights, 2022).

In the sense of the legal framework, Yakhin et al. (2017) describe a discrepancy among various countries, states, and regions regarding the categorization and registration of biostimulant products, which can impact trade hinder further developments. The terminologies associated with biostimulants in legislation include planting conditioners, other fertilizers, supplements, soil correctives, plant fortifiers, and phytofortifiers, for example. In addition to the terminology used for product registration, many jurisdictions require detailed information identifying all substances present in the product, while others allow registration without complete identification, noting that these products are considered matrices of complex composition (Yakhin et al., 2017).

In the European Union, for example, the last regulation from 2019 stipulates that biostimulants are fertilizer products that confer: (a) nutrient use efficiency, (b) tolerance to abiotic stress, (c) quality traits, (d) availability of confined nutrients in soil or rhizosphere. Products must clearly indicate on the label the declared effects tested on plants, the physical form, production and expiration dates, methods of application, and other relevant instructions, such as soil management practices, chemical fertilization, and incompatibility with plant protection products. In addition, the regulation sets limit values for contaminants such as cadmium, chromium, lead, mercury, nickel, and arsenic (EU, 2019).

In the United States, the regulation of biostimulants still has significant gaps, with no clearly defined legislation despite a gradual increase in the commercialization of these products. In 2018, the Farm Bill, approved as the Agricultural Improvement Act, established some delimitations regarding biostimulants. According to the regulation, products must be evaluated for their active components; thus, a plant biostimulant could be defined as a plant regulator under the Federal Insecticide, Fungicide and Rodenticide Act (FIFRA), transferring its regulation to the Environmental Protection Agency (EPA). Other biostimulant products, in turn, can be regulated by state departments of agriculture, which legislate on fertilizers, plant and soil amendments, and others that are not included in FIFRA (Madende and Hayes, 2020).

In Brazil, biostimulants are considered bioinputs and are part of the National Bioinputs Program (Brazil, 2020). In Bill No. 3,668 of 2021, biostimulants are defined as products containing microorganisms, metabolites of microorganism action, or organic components, either isolated or combined, that stimulate biological processes and must be registered with the Ministry of Agriculture, Livestock, and Supply. However, the legislation lacks clarity regarding the type of labeling required for the commercialization of the biostimulant (bioinput) in the Brazilian market (Brazil, 2021).

Finally, one of the biggest obstacles to the standardization of biostimulant products regarding their production, registration, marketing, and use is the large number of molecules in their composition that are claimed to have stimulant properties for plant development, as well as the differing regulatory requirements. Because of this, different countries have sought to harmonize production and evaluation processes in the search for a common standard for these bioactive molecules (Caradonia et al., 2019).

## 4 Compounds of interest

Over the years, researchers have proposed different categorizations of biostimulant products, especially based on the main component or modes of action. In European Union countries, e.g., this information is included on the label of commercialized products and is serves as a means of registering biostimulants. The classification based on the origin of the raw material is the most commonly used (Bulgari et al., 2019). Thus, biostimulants can be divided into two groups: those of biological origin, where the components and molecules come from the plant or other organic sources, and those of physical or chemical origin (Shahrajabian et al., 2021).

In this context, certain groups of compounds can be mentioned, including phytohormones, polyamines, amino acids, polyphenolic compounds, and polysaccharides (Ali et al., 2021; Garg et al., 2024; Gholizadeh et al., 2024; Kumar et al., 2024; Mughunth et al., 2024). Given the significance of these compounds, the literature review conducted by Mughunth et al. (2024) describes various biological activities of marine algae for use as biostimulants in plants. These activities include increased productivity and quality of agricultural crops, enhancement of biochemical compounds, improved yield and essential oil content, as well as increased tolerance to (a)biotic stress factors. Similarly, plant biostimulants can also achieve comparable results (Sible et al., 2021; Garg et al., 2024). Thus, it is evident that these substances possess considerable potential for enhancing plant growth and resilience.

Regarding phytohormones, these low-molecular-weight compounds play crucial roles in regulating plant growth and development, being associated with both morphophysiological and biochemical responses (Roychoudhury and Aftab, 2021; Aguilar-Ayala and Herrera-Rojas, 2023). One of the main phytohormones is abscisic acid (ABA), which has long been considered the primary phytohormone linked to stomatal regulation and plant stress responses. However, other phytohormones also play significant roles in plants, including cytokinins, auxins, gibberellins, and ethylene (Roychoudhury and Aftab, 2021).

ABA, a bioactive terpenoid phytohormone, is recognized as one of the primary components in seaweed extracts (Yalçın et al., 2019). Its significance in plant physiology arises from its pivotal role in regulating various stress responses and developmental processes. As a biostimulant, ABA enhances several biochemical parameters, such as chlorophyll content, antioxidant enzyme activity, and osmotic adjustment compounds, which collectively contribute to improved plant resilience under adverse conditions (Souza et al., 2013; Marcińska et al., 2013; Ghassemi et al., 2019).

Cytokinins, along with auxins, are responsible for complex biochemical processes within plant systems, although many of these processes remain to be fully elucidated (Sakakibara, 2021). Together with auxins, they are well-known for their roles in plant growth and are commonly found in macroalgae, where they, along with macro and micronutrients, enhance developmental processes in plants (Patel et al., 2018; Kalasariya et al., 2021). Additionally, the review by Cortleven et al. (2019) reports its action in plant defense mechanisms against light, temperature, drought, osmotic, salinity, and nutritional stress factors, as well as a complete response to certain plant pathogens and herbivores.

Auxins constitute a group of low-molecular-weight phytohormones essential for plant growth and development. These molecules regulate multiple physiological processes, including cell division, elongation, differentiation, organogenesis, and responses to environmental stimuli (Gomes and Scortecci, 2021). The most abundant and biologically active auxin is indole-3-acetic acid (IAA), although other naturally occurring auxins, such as indole-3-butyric acid (IBA), 4-chloroindole-3-acetic acid (4-Cl-IAA), and phenylacetic acid (PAA), also contribute to plant development (Cao et al., 2019). In addition to growth regulation, auxins play a key role in stress responses, particularly in modulating root architecture under adverse conditions, such as low temperatures (Tiwari et al., 2023).

Gibberellins are classified as carboxylated diterpenoids, with modifications in their chemical structure depending on their source. Their biosynthesis occurs through the methylerythritol 4-phosphate (MEP) pathway, and they are primarily found in higher plants (Lichtenthaler et al., 1997). The most abundant and the first gibberellin to be characterized was the gibberellic acid (i.e., gibberellin A3), derived from the fungus *Fusarium fujikuroi* (Curtis and Cross, 1954). This compound is still produced on an industrial scale for use in agriculture (Rademacher, 2016). Notably, among their various biochemical properties, these molecules in biostimulants contribute to hormonal regulation (Achard et al., 2008). Gibberellins, due to their interactions with other phytohormones, help maintain a balance in the physiological state of plants during both abiotic and biotic stress (Castro-Camba et al., 2022; Li et al., 2021).

Ethylene, in turn, is one of the simplest and most accessible molecules for biosynthesis in plant systems. Recognized as an essential growth regulator, ethylene is involved in a variety of physiological and developmental processes, ranging from growth regulation to the induction of fruit ripening, as well as in multiple stress responses (Chen et al., 2021a). In response to biotic stress, ethylene plays a role in disease resistance by inducing the expression of genes involved in plant defense, such as pathogen-related proteins (PRs) and phytoalexins (Jasrotia and Jasrotia, 2022). Moreover, Chen et al. (2021a) have shown that ethylene content is augmented in responses to abiotic stress factors, such as drought, flooding/hypoxia, osmotic pressure, salinity, heat, cold, and heavy metals.

In the group of plant growth regulator compounds, polyamines play an important role. These small, naturally occurring organic polycations, with contain two or more amino groups, are found in both prokaryotes and eukaryotes, and they are crucial for various physiological processes in plants, such as growth and the increase of bioactive compounds (Blázquez, 2024; Gholizadeh et al., 2024). They have also demonstrated the ability to act in stress responses (Shao et al., 2022; Gholizadeh et al., 2024). Among the main exogenous polyamines used as potential biostimulants are putrescine, spermidine, spermine, and termospermin (Blázquez, 2024).

Putrescine is a simple polyamine composed of two amino acids, ornithine and arginine. It plays a crucial role in cell growth, differentiation, and apoptosis. Putrescine is involved in various physiological processes, including stress responses and the regulation of gene expression in plants. In addition to its direct relation to plant growth and developmental processes, it contributes to tolerance against various abiotic stresses, such as salinity, low and high temperatures, and drought (González-Hernández et al., 2022).

Spermidine is a higher-order polyamine derived from putrescine. It is involved in cellular processes such as cell proliferation, differentiation, and apoptosis. Additionally, spermidine has been linked to the regulation of ion channels and is recognized for its antioxidant properties, which contribute to stress tolerance in plants (Li et al., 2022; Xie et al., 2023).

Spermine is another polyamine formed from spermidine. It has a more complex structure and is involved in various cellular functions, including protection against oxidative stress and regulation of ion transport. Spermine also plays a significant role in plant growth and development (Hasan et al., 2021; Hai et al., 2022). Furthermore, it serves as a regulator of biosynthetic pathways, decreasing ethylene levels while increasing phenolic compounds and sugars. This modulation enables plants to better tolerate harmful abiotic effects, such as drought and salinity (Silva et al., 2023).

Thermospermine is a polyamine derivative of spermine, particularly important in certain plant species and associated with plant growth-related genes (Kakehi et al., 2008; Furumoto et al., 2024). Additionally, it has been linked to adaptation to environmental stresses (Furumoto et al., 2024).

Amino acids are essential for plant metabolism, serving not only as building blocks for proteins but also as key regulators of stress responses. Proline aids in osmotic adjustment and provides protection against oxidative stress, while glutamate acts as a precursor for proline and GABA (γ-aminobutyric acid), both crucial for stress adaptation (Gramazio et al., 2020). Arginine contributes to nitrogen storage and oxidative stress signaling, whereas methionine and cysteine help regulate redox homeostasis through glutathione and polyamines (Acheampong et al., 2024). Lysine catabolism produces proline and pipecolic acid, enhancing plant stress tolerance (Arruda and Barreto, 2020), while tryptophan is vital for auxin biosynthesis and root development. These amino acids interact with phytohormones like ABA and ethylene, influencing drought resilience and salinity responses (Soda et al., 2022). Advances in metabolic engineering have targeted amino acid biosynthesis to improve stress tolerance, revealing both benefits and metabolic trade-offs in crop improvement.

Polyphenolics are a class of secondary metabolites that play crucial roles in plant metabolism. They are widely distributed throughout various plant parts, including leaves, flowers, fruits, and seeds (Zagoskina et al., 2023). A broad diversity of polyphenols exists, and these compounds positively affect plant growth and development while also serving as elicitors of tolerance to (a)biotic stress factors. Examples include salicylic acid (Song et al., 2023), anthocyanins (Li and Ahammed, 2023), gallic acid (Zhang et al., 2022a), quercetin (Jańczak-Pieniążek et al., 2021), and the stilbene resveratrol (Wagner et al., 2022).

Finally, polysaccharides are macromolecules formed by long chains of monosaccharides, which are simple sugars. In plants, these compounds play essential structural and functional roles, serving as fundamental components of plant cell walls (Kabir et al., 2022). The application of polysaccharides derived from both marine and terrestrial sources has shown significant positive responses in plants (González-Pérez et al., 2018; Mamede et al., 2023). The study by Mamede et al. (2023) indicates that alga-derived polysaccharides such as agar, alginate, and carrageenan can enhance seed germination and plant vigor, increase nutrient absorption from the soil, and protect plants against various abiotic and biotic stresses, including salinity, drought, temperature extremes, and pathogens. Similarly, plant-derived polysaccharides like as xyloglucan, found in various plant species, contribute to soil aggregation and the maintenance of its physical properties (Galloway et al., 2018). Under stress conditions such as salinity, xyloglucans can induce the expression of tolerance genes, mitigating the negative effects of oxidative stress in plants (González-Pérez et al., 2018).

## 5 Adverse weather conditions and impact on agriculture

Global warming, industrialization, and climate change are interconnected through complex relationships. Significant increases in greenhouse gas (GHG) emissions, mainly carbon dioxide (CO_2_), from the burning of fossil fuels (Shahbaz and Sinha, 2019; Ahmed et al., 2022), have occurred since the beginning of industrialization. The increase in CO_2_ concentrations and other GHGs in the atmosphere traps heat and contributes to global warming, altering climate events such as droughts, more frequent and intense heat waves, changes in precipitation patterns and temperatures, and the melting of glaciers, as well as rising temperatures and sea level (Rummukainen, 2013; Shahgedanova, 2021; Ballinger et al., 2023).

Such changes can directly impact crop production due to the adverse environmental conditions which plants will be exposed (Warsi et al., 2021).

Climate plays a crucial role in determining the success or failure of agricultural practices and overall crop productivity. Climatic conditions such as temperature, sunlight, and rain directly influence the growth and development of plants, as each crop has specific temperature and photoperiod requirements for optimal growth (Assad et al., 2013). These variables affect the progression of crop stages across a wide spectrum, encompassing both vegetative and reproductive phases (flowering and fruiting). Deviations in climate patterns can disrupt these phenological stages (Körner, 2021). Furthermore, precipitation impacts water availability by altering soil moisture levels, with insufficient or excessive precipitation leading to droughts or floods, respectively, which consequently influences the availability of nutrients that plants can absorb (Dhaliwal et al., 2022; Elbasiouny et al., 2022) According to the FAO (2015; 2023), extreme adverse weather events such as droughts, floods, heat waves, and cold spells significantly impact agriculture and affect crop yields. In summary, climate change threatens global crop production, requiring adaptive agronomic strategies to enhance resilience (Zuma et al., 2023). Among these strategies, biostimulants play a key role in mitigating stress by modulating plant physiology and improving tolerance to environmental challenges (Bhupenchandra et al., 2022).

### 5.1 Cold stress

Temperature plays a fundamental role in the growth and development of plants, and extremely low or high temperatures cause great stress, restricting natural processes at the genetic, biochemical, and physiological levels. Especially in recent decades, due to climate change this environmental factor has been widely discussed by experts in the field of food production, who seek to explore new possibilities to face this challenge. In fact, low temperatures have represented one of the main environmental factors for the suppression of agricultural crops, which leads to drastic losses annually in the world (Saleem et al., 2021).

Estimates indicate losses of 50% in the field due to plant stresses resulting from temperature changes (Sangiorgio et al., 2020). In 1972 and 1976, losses of 42% and 37%, respectively, were reported in rice production in the northeast region of China due to severe cold stress (Chen et al., 2021b). In horticulture, for example, countries such as France, Germany, Italy, Belgium, Switzerland, and the USA recorded large agricultural losses due to increased frosts in the past years (Sangiorgio et al., 2020).

Regarding the low temperatures, plants can be categorized as cold-sensitive, cold-tolerant, and freeze tolerant. Plants sensitive to cold are those that die at the beginning of stress conditions, not resisting extreme conditions. In turn, freezing-tolerant plants are those that face the greatest cold stress (Saleem et al., 2021). Due to their cold adaptation characteristics, the geographic distribution of plants is directly affected, where tropical and subtropical plants (e.g., rice, soybeans, tomatoes, corn, and cotton) are sensitive to cold stress, lacking the ability to acclimate to cold, as temperate species (e.g., barley, wheat, and rye) have a greater capacity for freezing tolerance (Ritonga and Chen, 2020).

Low temperatures above freezing conditions can slow plant metabolism, decreasing photosynthetic levels, leaf growth, and early senescence. In conditions lower than freezing, the development of buds can be seriously impaired, resulting in the destruction of rehydrated buds. In cases of frost, plant tissues dehydrate, resulting in an increase in the concentrations of osmolytes in the cell cytoplasm, consequently, resulting in the rupture of the plasma membrane (Sangiorgio et al., 2020). Generally speaking, damage to plant tissues can lead to poor growth, delayed flowering, reduced fruit set, and lower productivity overall. The severity and duration of cold stress can determine the extent of yield and quality losses and, in extreme cases, result in plant death or total crop loss, which can extend beyond the immediate growing season and affect the production of future crop cycles (Bhattacharya, 2022).

Plants respond to cold stress through several physiological mechanisms to deal with adverse conditions, namely changes in gene expression, cellular metabolism, and adjustments in water and nutrient absorption. The plant’s response can occur through different metabolic pathways, each of which can represent tolerance, attempts to avoid, escape, and recover (Chen et al., 2021b). For example, as a response to cold stress, several pathways can be affected, such as calcium channels (Ca^2+^). From the moment extracellular ice is formed, which leads to water loss in cells, the integrity of cell membrane systems is affected, leading to the opening of Ca^2+^ channels, due to the loss of membrane fluidity. The change in Ca^2+^ concentration induces the effect of signaling cascades through mitogen-active protein kinases (MAPKs), which can reach transcriptomic levels. In the carbohydrate pathway, in turn, immediate reprogramming occurs, as it is necessary to avoid any type of imbalance that could cause cell damage or death (Fürtauer et al., 2019). Thus, the effects of cold stress include physiological adaptations to water deficiency, such as the accumulation of various osmolytes and antioxidants, changes in phospholipid composition, production of reactive oxygen and nitrogen species, and the activation of phosphoprotein cascades. In fact, the knowledge about stress detection and plant response is a key issue so that preventive measures can be implemented at these times (Chen et al., 2021b).

In this sense, biostimulant elicitors can play a crucial role in increasing plant resilience and improving tolerance to cold stress, as they can activate the plant’s natural defense mechanisms against stresses, stimulating them to grow. Biostimulants can help in the production of protective compounds, such as antioxidants, osmoprotectors, and heat shock proteins, which help the plant to cope with the cold, and modulate gene expression in plants, activating specific genes associated with cold tolerance, increasing the plant’s ability to withstand low temperatures and reduces the negative impact of cold stress. Furthermore, biostimulants can improve various physiological processes in plants, including photosynthesis, respiration, and nutrient metabolism, which improve overall health and vigor, making them more resistant to cold stress (Askari-Khorasgani et al., 2019).

## 6 Mechanisms of action of biostimulants and biofertilizers

**Figure 1:**
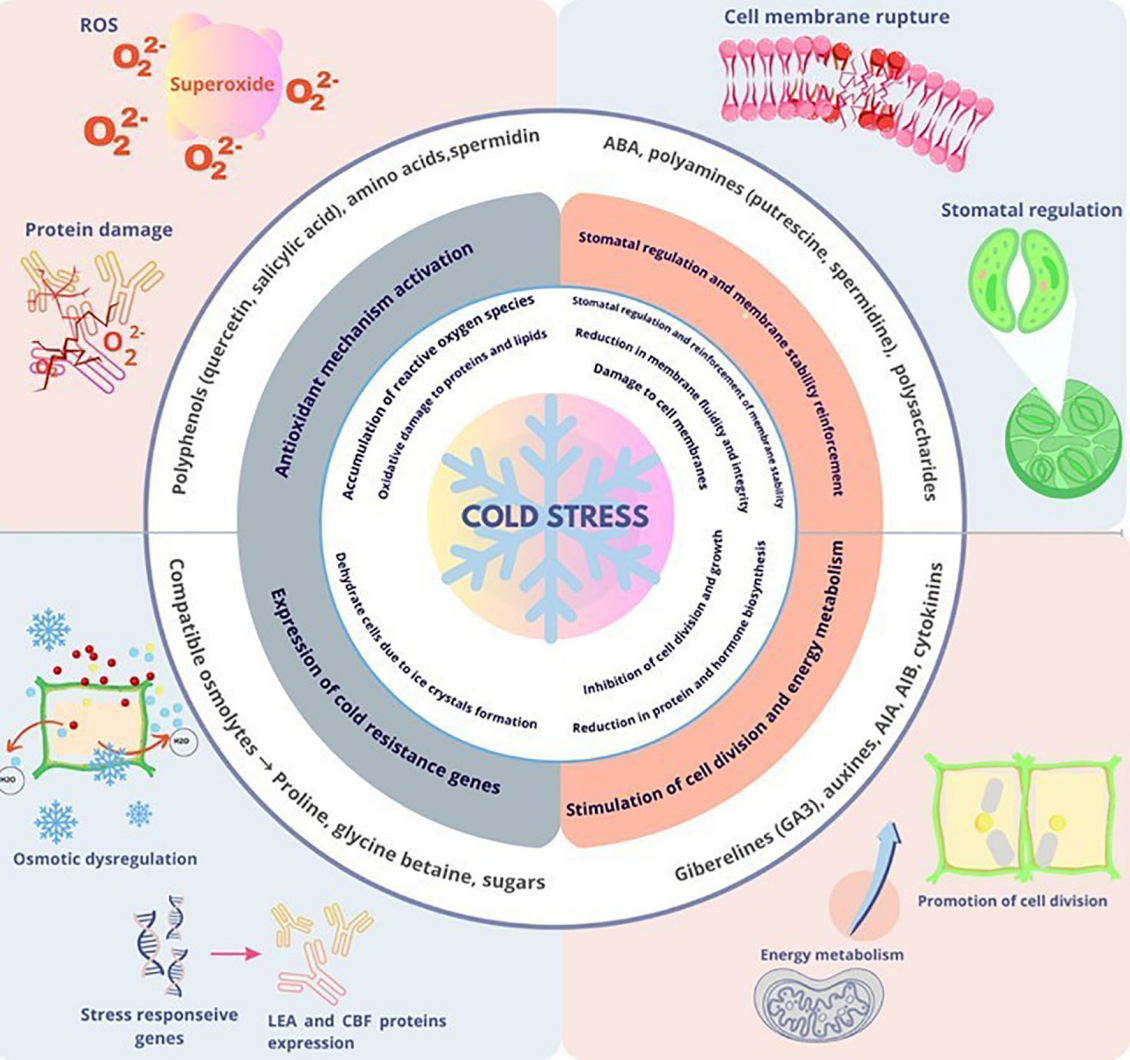
Plant responses to cold: damage, bioactive compounds, and tolerance mechanisms.

Biostimulants enhance plant tolerance to cold stress by activating multiple protective pathways (Zulfiqar and Ashraf, 2021; Martínez-Lorente et al., 2024; Navarro-Morillo et al., 2024). These compounds operate through three primary mechanisms: enhancing antioxidant defense systems, accumulating osmoprotectants, and modulating hormonal signaling (**Figure 1**). Together, these mechanisms maintain membrane integrity, stabilize cellular proteins, and sustain essential metabolic functions, thereby mitigating the detrimental effects of cold temperatures on plant physiology (Dreyer and Dietz, 2018; Monterisi et al., 2024a; van Oosten et al., 2017).

### 6.1 Antioxidant defense enhancement

Reactive oxygen species (ROS) are byproducts of cellular metabolism that can accumulate under stress conditions, such as extreme temperatures. While low levels of ROS function as signaling molecules, excessive accumulation leads to oxidative damage to lipids, proteins, and DNA (Demidchik, 2017). Plants possess sophisticated antioxidant defense systems to maintain ROS homeostasis (Nadarajah, 2020), and the redox-regulatory network is critical for cold stress acclimation (Dreyer and Dietz, 2018).

Biostimulants significantly enhance these natural defense mechanisms by increasing the activities of antioxidant enzymes, including superoxide dismutase, catalase, and ascorbate peroxidase, which are crucial for scavenging ROS (Cerruti et al., 2024; Vasconcelos, 2020). Navarro-Morillo et al. (2024) demonstrated that algae-based biostimulants improved the physiological condition of zucchini plants under cold stress, contributing to the limitation ROS accumulation while simultaneously providing osmoprotection.

### 6.2 Osmoprotectant accumulation

Osmoprotection is a critical mechanism through which biostimulants enhance plant tolerance to cold stress. Under low-temperature conditions, plants face cellular dehydration and potential ice crystal formation, which can damage cellular structures. Biostimulants stimulate the biosynthesis and accumulation of key osmolytes, including secondary metabolites (proline, betaine, and putrescine) and compatible solutes (sucrose, glucose, raffinose, fructose, and trehalose) (Jahed et al., 2023).

This elevated concentration of osmolytes within the cytoplasm maintains a lower cellular water potential than the external environment, even under cold-induced dehydration. The resulting osmotic adjustment promotes water influx into cells, preserving cellular hydration and turgor pressure – both essential for cell expansion, stomatal regulation, and overall metabolic function (Jiménez-Arias et al., 2021; Aloui et al., 2023). Additionally, these osmolytes interact directly with proteins and membranes, preventing denaturation and maintaining the functional integrity of essential cellular components, including metabolic enzymes, photosynthetic complexes, and membrane-bound transporters (Rydeen et al., 2018; Jahed et al., 2023).

### 6.3 Hormonal signaling modulation

Biostimulants exert significant effects on plant hormone pathways that regulate cold stress responses. Cold stress directly impacts auxin transport by inhibiting the recycling of transmembrane proteins responsible for hormone redistribution, which compromises the establishment of auxin gradients essential for regulated growth (Rahman, 2013). While the relationship between exogenous auxin application and cold tolerance remains incompletely understood, research suggests that auxins mediate plant responses to low-temperature stress. For instance, IAA stimulates arbuscular mycorrhizal development, potentially contributing to plant adaptation under adverse environmental conditions such as cold (Kunkel et al., 2024).

Tiwari et al. (2023) demonstrated that disruptions in auxin transport and signaling pathways alter root morphology under cold stress, characterized by reduced auxin levels in root tips. This reduction inhibits positive cell cycle regulators while enhancing negative regulators. Notably, exogenous auxin application promotes root growth under cold stress conditions. However, it does not fully restore normal growth, suggesting the involvement of additional factors in the comprehensive cold stress response.

Cytokinins (CKs) represent another crucial hormone class modulated by biostimulants. Prerostova et al. (2021) investigated the impact of CK levels on cold stress responses using *Arabidopsis thaliana* transformants with either enhanced biosynthesis (DEX: IPT) or increased degradation (DEX: CKX) of Cks. Under various light conditions, plants with elevated CK and auxin levels, along with increased salicylic acid concentrations, demonstrated enhanced stress acclimation. Conversely, plants with reduced CK and auxin contents displayed weakened stress tolerance. This study highlighted the critical interplay between CKs and light signaling in regulating cold stress responses. Biostimulant application stimulates cytokinin modulation, effectively improving plant responses to the physiological stress caused by low temperatures (Benito et al., 2024; Monterisi et al., 2024b).

Ethylene, another plant hormone implicated in stress responses, exhibits context-dependent effects on cold stress tolerance (Huang et al., 2023). While it can contribute to acclimation, excessive ethylene accumulation can intensify cellular damage under cold conditions. Biostimulants help maintain optimal ethylene levels, preventing the potentially detrimental effects of low temperatures on cell physiology (Navarro-Morillo et al., 2024).

## 7 Biostimulants with defense-inducing effects against low temperatures

### 7.1 Commercial products from aquatic environments

As a marine representative, the brown macroalga *Ascophylum nodosum* is the species most used in commercial biostimulant products (Fleurence, 2022). Products derived from *A. nodosum* (alone or together with other bioactive compounds) have been tested for their ability to induce chilling and freezing tolerance in plants (**Table 1**).

**Table 1:** Biostimulants of aquatic sources inducers of chilling and freezing tolerance in plants.

Biostimulant	Source	Tested plant	Temperature	Effect	Reference
Acadian^®^	Powdered alkali extract of *Ascophyllum nodosum*	*Arabidopsis thaliana*	-7.5°C/-5.5°C	Reduced 70% chlorophyll damage due to decreased expression of the chlorophyllase genes	Rayirath et al. (2009)
	Powdered alkali extract of *Ascophyllum nodosum*	*Arabidopsis thaliana*	-2°C	Augmented chlorophyll content, possibly due to the downregulation of chlorophyll degradation genes, and upregulation in genes encoding cryoprotection of chloroplast stromal protein	Nair et al. (2012)
	Powdered alkali extract of *Ascophyllum nodosum*	Tobacco (*Nicotiana tabacum*)	0°C, -3°C, -5°C	Improved cell growth, membrane stability, and nuclear integrity, and reduced cell death, due to the upregulation of key freezing tolerance genes	Zamani-Babgohari et al. (2019)
Algfect ^®^ and Algavyt Zn/Mn^®^	*Ascophyllum nodosum*, *Fucus* spp., *Laminaria* spp.	Maize (*Zea mays*)	12–14°C	Reduction of leaf necrosis, positive effects on plant growth and root development due to the increased superoxide dismutase activity	Bradáčová et al. (2016)
Garlic oil + Cytolan Star^®^	Garlic (*Allium sativum*) and powdered extract of *Ascophyllum nodosum*	Orange fruits (*Citrus* × *sinensis*)	5 ± 1°C	Induces lower percentage of fruit decay, total acidity and highest total sugar content	Kamel (2014)

The application of the commercial product Acadian^®^ (a powdered alkaline extract of *A. nodosum*) in *Arabidopsis thaliana* significantly increased tolerance to freezing temperatures both in both *in vitro* and *in vivo* assays. Extract-treated plants recovered from freezing temperatures of -7.5°C *in vitro* and -5.5°C in *in vivo*. The plants exhibited reduced expression of the chlorophyllase genes AtCHL1 and AtCHL2 during freezing stress, resulting in a 70% reduction in chlorophyll damage (Rayirath et al., 2009). This product also protected *A. thaliana* plants from induced cold stress (-2°C) by enhancing chlorophyll content, possibly due to downregulation of chlorophyll degradation genes (AtCLH1 and AtCLH2) and the upregulation of the transcription factor DREB1A and the COR78/RD29A genes, which encode cryoprotective chloroplast stromal protein – key regulators of cold stress tolerance (Nair et al., 2012).

Acadian^®^ also improved the survival of *in vitro* tobacco cells (BY-2) after exposure to freezing temperatures (0, -3, and -5°C). The treatment enhanced cell growth, membrane stability, and nuclear integrity, while reducing cell death of cold-stressed BY-2 tobacco cell lines. This seaweed extract influenced cellular and molecular regulation, triggering mechanisms such as osmolyte accumulation and antioxidant activity to combat freezing stress. This response was associated with the upregulation of key freezing tolerance genes, including galactinol synthase 2, pyrroline 5-carboxylate synthase, and acetyl-CoA carboxylase (Zamani-Babgohari et al., 2019).

Another alkaline powdered extract, the commercial products Algafect^®^ and Algavyt+Zn/Mn^®^ (a combination of the brown seaweeds *A. nodosum*, *Fuccus* spp., and *Laminaria* spp.), demonstrated positive effects on maize plants subjected to low temperatures (12 – 14°C). The combination of these two products reduced leaf necrosis in maize (with only 0–15% of the leaf area affected) and promoted plant growth, particularly in root development, as observed with Algavyt Zn/Mn^®^. The beneficial effect of the Zn/Mn treatments and seaweed extracts were associated with increased superoxide dismutase (SOD) activity in the root and leaf tissues, playing a key role in defense against oxidative stress, with Zn, Mn, Cu, and Fe serving as essential enzymatic co-factors (Bradáčová et al., 2016).

The use of a product combining *A. nodosum* extract (Cytolan Star^®^) with garlic oil improved the quality of Valencia orange fruits during cold storage at 5°C. Specifically, a solution of 1 g/l Cytolan Star^®^ extract combined with 0.5% garlic oil resulted in the lowest percentage of fruit decay, the smallest reduction in total acidity, and the highest total sugar content. Additionally, treating the fruits with a mixture of 0.5% or 1% garlic oil and 3 g/l Cytolan Star^®^ led to the lowest loss of weight (%) (Kamel, 2014).

### 7.2 Commercial products from terrestrial sources

Biostimulants from terrestrial sources (e.g., plant extracts, agricultural residues, soil microorganisms, and animal protein hydrolysates) have also been tested in plants to induce tolerance to low temperatures (**Table 2**).

**Table 2:** Biostimulants derived from terrestrial sources inducers of chilling and frost tolerance in plants.

Compounds	Source	Tested plant	Temperature	Effect	Reference
Biozyme^®^ and humic acid	Fermented compounds of vegetables, melted rice, seaweed, yeast extract and minerals	Leek (*Allium ampeloprasum*), Celery (*Apium graveolens*), and Parlsey (*Petroselinum crispum*)	10 and 15°C	Increased seed germination	Yildirim et al. (2013)
Karrakin (KAR)	Smoked wheat straw	Tomato (*Solanum lycopersicum*)	–	Improved tomato growth and yield by regulating nutritional uptake, leaf temperature, photosynthesis, ROS scavenging, and CBF transcriptional activation	Liu et al. (2023)
Pepton 85/16^®^	Enzymatic hydrolysis of porcine hemoglobin - PHH	Lettuce (*Lactuca sativa*)	-3°C	Increased mean fresh and dry weights	Polo et al. (2006)
	Enzymatic hydrolysis of porcine hemoglobin - PHH	Tomato (*Solanum lycopersicum*)	11.0°C to 12.6°C	Improved root growth due to the extra amino acid availability and the biosynthesis of salicylic acid	Casadesús et al. (2020)
Pepton 85/16^®^ and Siapton^®^	Enzymatic hydrolysis of porcine hemoglobin - PHH	Strawberry (*Fragaria* × *ananassa*)	-6°C	More biomass of newly formed roots, earlier flowering and fruit production	Marfà et al. (2009)
Terra-Sorb^®^ foliar	Amino acid product obtained by enzymatic hydrolysis	Lettuce (*Lactuca sativa* L. var. capitata)	Root zone: 6°C, and diurnal colds: 4°C	Higher recovery of fresh weights, increased plants weight, stomatal opening and transpiration	Botta (2013)
	Amino acid product obtained by enzymatic hydrolysis	Maize (*Zea mays*)	10°C	Increased plant performance: leaf gas exchange, photosynthetic pigment content, and chlorophyll fluorescence	Cholakova-Bimbalova et al. (2019)
Sorghum water extract (SWE), Moringa leaf extract (MLE), Salicylic acid and thiourea	*Sorghum* sp. and *Moringa* sp.	Maize (*Zea mays*)	8-13°C	Increased crop growth rate, leaf area index, leaf area duration, plant height, grain yield, and total dry matter accumulation	Waqas et al. (2017)
Root endophytic fungus	*Piriformospora indica*	*Arabidopsis thaliana*	-6°C/4°C	Reduced negative effects of freezing, increased the amounts of soluble proteins, proline and ascorbic acid and stimulated the expression of several genes involved in the CBF-dependent pathway	Jiang et al. (2020)
Frostburn^®^	Coffee refuse	Japanese pear cultivars (*Pyrus* spp)	−3°C	At 2 ppm delayed flower bud freezing by 8 hours (vs. 3 hours in controls) and preserved pollen germination under cold stress (−3°C) in sensitive cultivars (e.g., ‘Kosui’)	Shibasaki et al. (2023)

The commercial product Pepton 85/16^®^ (an enzymatic hydrolyzed porcine hemoglobin – PHH) was tested in crop species such as tomato, lettuce, and strawberry plants. In the lettuce experiment, the mean fresh and dry weights of plants subjected to intense cold (-3°C, for 4h) and then treated with PHH at concentrations of 0.4, 0.8, and 1.6 mg/mL H_2_O were significantly greater than those of untreated plants (Polo et al., 2006). In tomato plants, Pepton (430 mg PHH/container) in a hydroponic system at low temperature (water temperature between 11.0°C and 12.6°C) promoted root growth by day 4 of the experiment, likely due to increased amino acid availability for growth and/or stimulation of specific hormonal pathways, such as salicylic acid (Casadesús et al., 2020). Strawberry plants were exposed to nighttime temperatures below zero, reaching -6°C, showed that the highest concentration of PHH (4 L/ha) resulted in a greater biomass of newly formed roots compared to the 2 L/ha treatment. Furthermore, both PHH treatments (2 and 4 L/ha) stimulated early flowering and significantly enhanced initial fruit production (Marfà et al., 2009).

Other commercial products found to induce cold tolerance in plants include Terra-Sorb^®^ Foliar and Biozyme^®^. Terra-Sorb^®^ Foliar is a protein hydrolyzate rich in amino acids obtained through enzymatic hydrolysis. In lettuce, a foliar application of 3 mL/L led to a greater recovery of fresh weight in both the root zone (at 6°C) and during daytime cooling (at 4°C). Additionally, treated lettuce exhibited significantly higher stomatal conductance at the onset of cold stress, resulting in enhanced stomatal opening and increased transpiration, which promotes the flow of water and nutrients from roots to shoots - an effect likely due either to the direct action of amino acids on stomata or to improved plant water status via stimulated root growth (Botta, 2013). When applied to maize plants grown at 10°C, Terra-Sorb^®^ Foliar improved leaf gas exchange parameters by 22–23% and increased photosynthetic pigment content by around 15%, further supporting its potential to enhance recovery and overall cold tolerance in crops (Cholakova-Bimbalova et al., 2019).

In its turn, Biozyme^®^ is a fermented product of vegetables, seaweed, and yeast. When applied in combination with humic acid, it resulted in enhanced seed germination of leek, celery, and parsley across all temperature treatments, including 10°C and 15°C (Yildirim et al., 2013). In addition to commercial products, other plant extracts have also been tested for chilling and freezing tolerance (Waqas et al., 2017; Jiang et al., 2020). A mix of two plant extracts (sorghum and moringa) combined with salicylic acid and thiourea was tested in maize plants and demonstrated stress-alleviating effects by improving physiological and biochemical attributes, as well as overall maize growth, under suboptimal temperature stress (8-13°C) (Waqas et al., 2017). Furthermore, the co-cultivation of the endophytic root fungus *Piriformospora indica* with *A. thaliana*, which was exposed to -6°C for 6 h, showed reduced negative effects of freezing. The plants exhibited higher levels of soluble proteins, proline, and ascorbic acid during the post-thaw recovery period at 4°C for 12 h (Jiang et al., 2020).

Frostburn^®^, a coffee-derived extract with anti-ice-nucleation properties, was evaluated at 2 ppm for mitigating cold-induced damage in the Japanese pear cultivars (*Pyrus* spp.) ‘Kosui’ and ‘Hosui’. When applied during the scale-separation stage (from pollen mother cell to tetrad phase), the extract delayed the onset of flower bud freezing by 8h, compared to just 3h in water-treated controls. This intervention preserved pollen germination rates under low-temperature stress (−3°C for 10h), attributed to reduced cellular dehydration and membrane disruption. Notably, ‘Hosui’ cultivar exhibited inherent cold tolerance, maintaining germination rates even at -6°C, while Frostburn^®^ specifically enhanced resilience in the more sensitive ‘Kosui’ cultivar, highlighting its potential as a cost-effective commercial solution for cold stress management in orchards (Shibasaki et al., 2023).

An interesting group of natural plant growth regulators called karrakins (KAR), found in smoke from wheat straw, also has the ability to induce tolerance to low temperatures in plants (Liu et al., 2023). When applied alongside smoked water (SW) on tomato plants, KAR improved growth and yield under suboptimal temperature conditions by regulating nutrient uptake, leaf temperature control, photosynthetic defense mechanisms, reactive oxygen species scavenging, and the transcriptional activation of C-repeat binding factors (CBF). Thus, SW, which operates through the KAR-mediated strigolactones (SLs) and ABA signaling network, holds potential for enhancing cold tolerance in tomato production (Liu et al., 2023).

### 7.3 Non-commercial products

In addition to established commercial formulations, scientific research has increasingly explored naturally derived and non-commercial blends for their potential to enhance cold tolerance in plants (**Table 3**). A recent review by Akhtar et al. (2024) highlighted the use of nanobiotechnology and bacteria to promote cold tolerance in crop species. To better understand the current landscape of developing new products that induce cold tolerance in plants, it is relevant to expand the focus include other alternatives, such as crude algal extracts, plant-based extracts, and multi-component botanical and/or microbial mixtures. Although these formulations are not yet standardized or commercially available, they have been investigated for their ability to induce cold tolerance in crops.

**Table 3:** Non-commercial sources that induces cold or chilling tolerance in plants.

Biostimulant	Source	Tested plant	Temperature	Effect	Reference
Brown seaweed extract	Non-identified brown seaweed	Tomato (*Solanum lycopersicum*)	4°C	Higher stomatal conductance, net photosynthesis, and yield in plants treated with the biostimulant. Biochemical improvements were corroborated by molecular data	Borella et al. (2023)
Hot water	Water	Tomato fruits (*S. lycopersicum*)	5°C	The treatment with HWT (42°C/5 min) induced better metabolic performance of tomato fruits under cold storage. The reported effeect was associated with a higher accumulation of antioxidants and osmolytes	Delgado-Vargas et al. (2022)
CRDAP–ZNC composite	Diammonium phosphate and *Paecilomyces variotii* extract	Winter wheat (*Triticum aestivum* L.)	Near 5°C (most of the time in the first 10 days)	Enhanced cold tolerance, with 22% higher soil available phosphorus, 30% increased root growth, up to 17% higher photosynthesis, and 8.6% reduction in H_2_O_2_ content	Chen et al. (2023)
Moringa leaf extract	*Moringa oleifera*	Spring hybrid maize (*Zea mays* L.)	5°C-10°C	The seed priming enhanced stand establishment and reduced chilling damage. The 20-day-old transplanted seedlings showed improved agronomic traits, yield, and quality compared to direct sowing and 30-day-old transplants	Junaid et al. (2019)
*Trichoderma harzianum* seed inoculant	*T. harzianum* Rifai strain T-22 (American Type)	Tomato (*S. lycopersicum*) cv. Jubilee	10 ± 0.2°C	Accelerated germination speed of seeds under chilling stress	Mastouri et al. (2010)
	*T. harzianum* strain AK20G isolated from soil	Tomato (*S. lycopersicum* L.) cv. CaljN3	8°C	Inoculated plants restored the photochemical PS II efficiency, plant growth and electrolyte leakage percentage under cold stress. Relative expression rates of *Tas14* and *P5CS* genes were significantly higher in treated plants than control	Ghorbanpour et al. (2018)
	*T. harzianum*	Maize (*Zea mays* L.)	5°C	Seed priming enhanced cold tolerance, improved seedling emergence, root dry weight, and catalase activity, particularly in the cold-resistant cultivar AR68.	Afrouz et al. (2023)
Various fungal isolates	*Brassica oleracea* endophytic fungal	Kale (*B. oleracea* var. *acephala*)	12°C	Inoculation with *Phialocephala* sp., *Fusarium* sp., and *Acrocalymma* sp. increased plant growth and weight.	Poveda et al. (2020)

Studies have investigated the role of *Bacillus* spp. in mitigating cold stress, demonstrating their ability to enhance plant resilience through multiple mechanisms. For instance, cold-tolerant strains such as *Bacillus* GBAC46 and RJGP41 promote growth by increasing the IAA biosynthesis and the activity of enzymes like SOD, CAT, and APX. Additionally, their volatile organic compounds (VOCs) improve root development and upregulate stress-related genes (Khan et al., 2023). Similarly, *Lysinibacillus fusiformis* and *L.* sp*haericus* enhance phosphorus solubilization and osmolyte accumulation (proline and glycine betaine), which improves photosynthesis and membrane stability under low temperatures (Jha and Mohamed, 2023). Furthermore, the synergistic action of *Bacillus subtilis* and *Piriformospora indica* further strengthens cold tolerance by enhancing biomass production, enzymatic defenses, and stress-related gene expression (Shi et al., 2024). These findings highlight *Bacillus* spp. as key contributors to plant adaptation under cold stress conditions.

In the study by Borella et al. (2023), a biostimulant derived from an unspecified brown alga species was evaluated in tomatoes exposed to low temperatures. Physiological and molecular analyses were conducted, revealing that stomatal conductance, net photosynthesis, and yield were significantly higher in the plants treated with the biostimulant compared to the untreated plants. Additionally, the molecular analysis indicated that the extract resulted in increased cellular contents of proline, polyphenols, flavonoids, tannins, and carotenoids.

In a postharvest study, Delgado-Vargas et al. (2022) evaluated the effects of hot water treatment (HWT – 45°C/5 min) on tomato fruits under chilling conditions (5°C/20 days). HWT-treated fruits exhibited reduced chilling injury (CI), higher total phenolics (TP), and greater antioxidant activity (AoxA) compared to controls. They also showed increased accumulation of phenolics, sugars, and certain alkaloids, potentially mediated by azelaic acid, glutamine, and tryptophan. Contrarily. N-feruloylputrescine, esculeoside AII, and hydroxy-α-tomatine II contents decreased. The improved metabolic performance of HWT-treated tomatoes during cold storage was linked to the accumulation of antioxidant and osmolyte compounds. Identifying metabolites associated with CI reduction enhances the understanding of tolerance mechanisms and offers targets for CI prevention, such as genetic improvements or direct metabolite application.

Chen et al. (2023) investigated the cold tolerance response of winter wheat (*Triticum aestivum* L.) treated with a composite of controlled-release diammonium phosphate (CRDAP) and the extract of the fungus species *Paecilomyces variotii* (ZNC). Under spring low-temperature stress, the composite synchronized phosphorus release with crop demand, boosting soil available phosphorus by 22% in the top 40 cm, enhancing root growth by nearly 30%, and elevating photosynthetic rates by up to 17% compared to conventional fertilizers. Moreover, there was an increased biosynthesis of IAA in roots and strengthened antioxidant defenses, with an 8.6% reduction in hydrogen peroxide (H_2_O_2_) concentration, further improving cold resilience.

Mastouri et al. (2010) investigated the effects of *Trichoderma harzianum* T22 seed treatment on tomato seeds under chilling stress. The results showed that T22-treated seeds germinated significantly faster than untreated seeds, even when exposed to chilling stress at 10°C for up to 3 days. Although T22 treatment accelerated germination speed, the final germination percentage did not differ significantly from that of the control seeds. This positive effect of T22 on germination under thermal stress suggests that the application of this fungal species may trigger a physiological response in seeds, enhancing germination speed under cold conditions without negatively affecting the final germination rate.

In the study conducted by Poveda et al. (2020), the inoculation of kale plants with various fungal isolates, including *Pyrenophora* sp., *Fusarium* sp., *Phialocephala* sp., *Chaetomium* sp., *Diaporthe* sp., and *Acrocalymma* sp., significantly increased plant growth and tolerance under cold stress (12°C). Inoculation with *Phialocephala*, *Fusarium* sp., and *Acrocalymma* sp. notably enhanced plant weight, with an almost twofold increase compared to the control. Similarly, dry weight also increased in the inoculated plants, highlighting the positive impact of fungal inoculation on cold tolerance.

Afrouz et al. (2023) determined the effect of seed biopriming with *Trichoderma harzianum* on maize tolerance to cold stress. The results showed that biopriming significantly enhanced seedling emergence and physiological parameters, particularly under cold conditions (5°C). Among the pretreatments, *T. harzianum* resulted in improved root dry weight and increased catalase activity in the maize cultivars investigated, with the highest root dry weight detected in the cultivar AR68. These findings suggest that *T. harzianum* priming effectively enhances tolerance in maize.

In the study by Ghorbanpour et al. (2018), the inoculation of *T. harzianum* AK20G strain in tomato seeds was investigated for its role inducing cold tolerance in plantlets exposed to 8°C for 6 days. The results showed that *T. harzianum* effectively mitigated the adverse effects of cold stress by enhancing photosynthesis and growth rates, reducing lipid peroxidation, and minimizing electrolyte leakage. Additionally, this fungal species improved leaf water content and proline accumulation while promoting the expression of genes involved in cold stress tolerance, such as TAS14 and P5CS.

Junaid et al. (2019) evaluated the effect of moringa leaf extract (MLE – *Moringa oleifera*) in maize (*Zea mays* L.) exposed to low temperatures (5°C-10°C) during early seedling development. Seed priming (24 h) with 3% MLE improved chilling tolerance by enhancing the stand establishment and increasing both emergence percentage and speed. The greatest improvements in agronomic traits, yield, and quality were observed in 20-day-old transplanted seedlings, while direct sowing and 30-day-old transplants showed inferior performance. These results highlight the potential of MLE priming to mitigate chilling stress and enhance maize productivity under temperature fluctuations.

### 7.4 Other exogenously applied cold and chilling tolerance inducers

Since commercial products or natural extracts that effectively induce tolerance to cold, chilling, or freezing effects are scarce, some isolated compounds with the intended biological effect have been investigated, yielding interesting results (**Table 4**).

**Table 4:** Compounds with biostimulant effect that induces chilling or frozen tolerance in plants.

Compounds	Tested plant	Temperature	Effect	Reference
ABA	Rice (*Oryza sativa*) and weedy rice (*O. sativa* f. *spontanea*)	5°C	Reduced the chilling damage by activating antioxidant enzymes	Wang et al. (2013)
	Grapevine (*Vitis vinifera*)	4°C and 14°C	Increase the expression of promote freezing tolerant proteins	Rubio and Pérez (2019)
	Mangrove (*Kandelia obovata*)	9.7°C, -4.1°C and -5.3°C	Alleviated the effects of freezing stress due to the enrichment of arginine and proline, starch and sucrose metabolism	Liu et al. (2022)
	Banana (*Musa* spp.) and tomato (*Solanum lycopersicum*)	4°C	Alleviated chilling injury by accumulation of endogenous ABA, unsaturated fatty acids, and flavonoid content, and reduced the saturated fatty acid content	Song et al. (2022)
ABA and GB	*Arabidopsis thaliana*	-3.1°C to -4.5°C	Increased freezing tolerance and endogenous glycine betaine (GB) content	Xing and Rajashekar (2001)
*GB*	Maize (*Zea mays*)	15°C	Improved germination scores, soluble sugars and antioxidants	Farooq et al. (2008)
	Tomato (*S. lycopersicum*)	14°C	Increased the seed germination scores and regulate gibberellin (GA) and abscisic acid (ABA)	Zhang et al. (2022b)
SA	Maize (*Zea mays*)	2°C	Upregulation of antioxidant enzymes	Janda et al. (1999)
	Tomato (*Solanum lycopersicum*)	5°C	Induced the synthesis of stress-related proteins	Ding et al. (2002)
	Maize (*Zea mays*)	5°C	Inhibition of catalase activity	Horváth (2002)
	Peach (*Prunus persica*)	0°C	Chilling tolerance by upregulation of a heat shock protein (sHSP) expression	Wang et al. (2006)
	*Anthurium andraeanum*	4°C and 12°C	Suppressed chilling injury by maintaining high contents of antioxidants and membrane integrity	Promyou et al. (2012)
	Lemon fruit *(Citrus limon)*	-0.5, 2°C and 4.5°C	Increased total phenolics and antioxidant enzyme activity	Siboza et al. (2014)
	Apple (*Malus domestica*)	8°C	Enhanced antioxidant enzymes expression and endogenous SA	Wang et al. (2016)
	Wheat (*Triticum aestivum*)	-7°C	Increased freezing tolerance by improved in photochemical efficiency, accumulation of osmo-protectant and antioxidant compounds	Wang et al. (2022)
	Maize (*Zea mays*)	4°C	Increased cold tolerance by lowering MDA concentration and increasing antioxidant contents, besides osmotic adjustment associated with augmented proline amounts	Zhang et al. (2021)
SA, AsA, and H_2_O_2_	Maize (*Zea mays*)	>2.9°C (early stages)	Improved seed growth, leaf relative water, chlorophyll *b* contents, membrane stability, and enzymatic antioxidant activities	Ahmad et al. (2014)
MT	Bermudagrass (*Cynodon dactylon*)	4°C/-5°C and -5°C	Increased cold tolerance by lowering MDA and electrolyte leakage (EL) and increase antioxidant enzymes	Fan et al. (2015)
	Maize (*Zea mays*)	5°C	Improved germination under cold stress, proteomic changes linked to stress tolerance	Kołodziejczyk et al. (2016)
	Waxy maize (*Zea mays*)	13°C	Enhanced seed vigor, root growth, antioxidant enzyme activity, and starch metabolism while reducing oxidative damage	Cao et al. (2019)
	Tea (*Camellia sinensis*)	4°C	Enhances cold tolerance by lowering MDA level, increasing photosynthetic efficiency, alleviating ROS burst and up-regulate expression of antioxidant enzyme	Li et al. (2019)
	Barley (*Hordeum vulgare*)	5°C and 15°C	Alleviate growth inhibition of seedlings, restored circadian rhythmic oscillation and lowers MDA and soluble sugars level	Chang et al. (2021)
PA	Wheat (*T. aestivum* L.)	4°C	Shoot and root dry weight, chlorophyll, carotenoid, proline contents, catalase and ascorbate peroxidase activities were improved.	Gholizadeh et al. (2024)
	Apple (*M. domestica*)	-10°C to -25°C	Spermidine improved cold tolerance by stabilizing tissues, reducing ROS effects through increased proline content and antioxidant enzyme activity, besides enhancing polyamine metabolism	He et al. (2024)

*ABA, Abscisic acid; GB, Glycine betaine; SA, Salicylic Acid; MT, Melatonin; AsA, Ascorbic Acid; H_2_O_2_, Hydrogen peroxide; PA, Polyamines; MDA, malondialdehyde.

The plant phytohormone ABA has been demonstrated to be a key regulator of plant responses to abiotic stresses, including chilling and freezing (Vishwakarma et al., 2017). ABA application reduced the chilling damage (5°C) in four rice genotypes. Pre-treatment with ABA decreased the levels of superoxide anion (O_2_^-^), H_2_O_2_ and malondialdehyde (MDA) caused by chilling stress through increasing the activities of SOD, CAT, APX, glutathione reductase and the contents of ascorbic acid and glutathione (Wang et al., 2013). ABA also alleviated chilling injury of banana fruits, by inducing the accumulation of endogenous ABA, (un)saturated fatty acids, and flavonoids, by upregulating the transcription levels of MaABI5-like, fatty acid desaturation genes, and flavonoid synthesis-related genes during cold storage (Song et al., 2022). The application of ABA (26 mg/mL) to dormant buds of grapevine (*Vitis vinifera*) increased the expression of the CBF/DREB1, VvCBF2, VvCBF3, VvCBF4, and VvCBF6 transcription factors (Rubio and Pérez, 2019). Liu et al. (2022) describe that applying ABA in the mangrove species *Kandelia obovata* under natural frost conditions at approximately 32°N latitude effectively alleviated the adverse effects of freezing stress. This was achieved by activating antioxidant enzyme activity and increasing the accumulation of osmolytes, such as proline. Notably, the effectiveness of these responses was proportional to the concentration of ABA applied.

Another interesting effect of the exogenous application of ABA is the increase in endogenous concentrations of glycinebetaine (GB) (Xing and Rajashekar, 2001). GB is an amino acid that helps protect plants against abiotic stresses through osmoregulation or osmoprotection, and it contributes to the differential expression of stress tolerance-related genes (Giri, 2011). When applied in isolation, GB has demonstrated various effects on cold or freezing tolerance in plants. For instance, a 10 mM GB foliar spray on *Arabidopsis tahaliana* increased the freezing tolerance of plants to -4.5°C (Xing and Rajashekar, 2001). Similarly, maize seeds treated with GB significant improvements in germination rate, root and shoot length, seedling fresh and dry weight, leaf and root scores, soluble sugars, α-amylase activity, and total antioxidants capacity compared to untreated seeds under both optimal (27°C) and stress conditions (15°C) (Farooq et al., 2008). GB-treated tomato seeds (10 mmol/L) also exhibited better germination rates under cold stress (14°C). Analysis of gene expression and metabolism revealed that GB positively regulated endogenous gibberellin content, while the opposite effect was observed for abscisic acid content. Moreover, GB reduced the starch content in the tomato seeds by upregulating amylase gene expression (Zhang et al., 2022b).

Another important compound with recognized tolerance-inducing activity in plants is SA (Khan et al., 2015). Under low-temperature stress conditions (2°C), the application of 0.5 mM SA led to the upregulation of antioxidant enzymes, including APX, SOD, guaiacol peroxidase (GPOX), and GR (Janda et al., 1999). It also inhibited the activity of isozymes CAT-1 and CAT-2 in *Z. mays* (Horváth, 2002). The application of SA at 50 mg/L on the leaves of *Z. mays* seedlings under 4°C increased the antioxidant activities of APX, CAT, SOD, and peroxidase (POD), which decreased relative electrolyte conductivity (REC) and the levels of MDA and ROS (H_2_O_2_ and O_2_^−^). Additionally, there was an increase in proline content and relative water content (RWC) in the maize seedlings, enhancing their osmotic adjustment capacity (Zhang et al., 2021). High activities of CAT and SOD were also found in cut anthurium flowers (*Anthurium andraeanum*) subjected to cold stress (4°C to 12°C), resulting in detoxification of ROS and maintenance of membrane integrity (Promyou et al., 2012).

In apple plants (*Malus domestica*), SA increased the expression of cytosolic malate dehydrogenase and improved the redox state of the plant cell. Furthermore, transgenic plants that overexpressed cytosolic malate dehydrogenase (MdcyMDH) demonstrated greater tolerance to cold stress compared to the wild type, as they produced higher amounts of free and total amino acids (Wang et al., 2016).

The application of SA also aids in post-harvest activities by increasing the chilling tolerance of fruits during cold storage through the upregulating of stress-related proteins. Low concentrations of methyl salicylate (MeSA), i.e., 0.01 mM, helped protect tomato fruits (*S. lycopersicum*) in cold storage (5°C) by inducing the synthesis of certain stress-related proteins, such as the PR proteins PR-2b and PR-3a mRNAs, while also slightly increasing PR-3b mRNA accumulation (Ding et al., 2002). MeSA treatment on peaches fruits (*Prunus persica*) during cold storage (0°C) helped overcome chilling stress by upregulating the expression of small heat shock protein (sHSP) genes, ultimately leading to the production of sHSP, particularly HSP17.6 (Wang et al., 2006). Furthermore, a concentration of 2.0 mM SA-mediated enhanced the synthesis of total phenolics and increased the activity of phenylalanine ammonia-lyase, thereby improving chilling tolerance in cold-stored lemon fruit (*Citrus limon*; Siboza et al., 2014).

The study by Wang et al. (2022) investigated the effects of cold and SA on the accumulation of osmolytes in wheat leaves under freezing stress. The results indicated that both treatments significantly increased the levels of sucrose and free proline, improving the leaf’s water potential and reducing cell death, which enhanced tolerance to freezing. The accumulation of proline was promoted by both increased synthesis and inhibition of its degradation. Additionally, cold and SA stimulated the synthesis and hydrolysis of sucrose, positively regulating glucose catabolism and ammonia assimilation. These findings suggest that both treatments favor the accumulation of proline and sucrose, coordinating carbon and nitrogen metabolism to confer resistance to freezing stress.

The combination of SA with other compounds has also yielded interesting results. When SA was applied alongside AsA and H_2_O_2_, it improved seedling growth, leaf relative water content, chlorophyll *b* levels, membrane stability, and enzymatic antioxidant activities in pot-cultivated maize (Ahmad et al., 2014). In field experiments, the application of these substances – either through seed priming or foliar spray – enhanced morphological and yield-related attributes, as well as grain yield of spring maize under suboptimal temperatures in the early stages (>2.9°C). Notably, seed priming proved to be more effective than foliar application (Ahmad et al., 2014).

Priming treatments have also been shown to induce cold tolerance in plants. For instance, melatonin (MT) priming in maternal wheat plants (*T. aestivum*) during the grain filling stage promoted seed germination at 10°C in offspring. This effect was attributed to accelerated starch degradation and improved cold tolerance of the seedlings (10°C day; 6°C night) through the activation of antioxidant enzymes and enhancement of photosynthetic electron transport efficiency (Li et al., 2018).

The application of MT has been linked to specific mechanism of cold tolerance, such as a decrease in MDA levels and electrolyte leakage (EL). Additionally, it has associated with increased amounts of chlorophyll and enhanced enzymatic activity (e.g., SOD and POX), as well as changes in 46 metabolites when exogenously applied to bermudagrass plants (*Cynodon dactylon*). Among the measured metabolites, five sugars (arabinose, mannose, glucopyranose, maltose, and turanose) and one organic acid (propanoic acid) were significantly increased (Fan et al., 2015). Tea plants (*Camellia sinensis*) treated with MT on their leaves exhibited a reduction in ROS burst, decreased MDA levels, and maintained high photosynthetic efficiency. Moreover, these tea plants demonstrated elevated levels of GSH and AsA, along with increased activities of SOD, POX, CAT, and APX under cold stress (4°C). Notably, MT treatments can positively upregulate the expression of genes involved in the biosynthesis of antioxidant enzyme, specifically CsSOD, CsPOX, CsCAT, and CsAPX (Li et al., 2019).

In a similar study, maize seed priming with MT (50 and 500 μM) enhanced stress tolerance by improving germination under suboptimal thermal conditions (i.e., 5°C) and modifying the seed proteome. Proteomic analysis revealed MT-induced changes in protein expression related to stress response, suggesting a biochemical mechanism for enhanced chilling tolerance. These findings indicate that MT priming can be an effective strategy to improve maize resilience under low-temperature conditions (Kołodziejczyk et al., 2016).

In another priming study, Cao et al. (2019) demonstrated that MT (50 and 100 μM) improved maize germination under chilling stress at 13°C by increasing germination potential, seed vigor, and root growth. MT-treated seeds exhibited reduced oxidative damage (lower H_2_O_2_ and MDA levels) and enhanced antioxidant enzyme activity (SOD, POX, CAT, APX), along with improved starch metabolism, further supporting its role in cold stress tolerance.

Additionally, the exogenous application of MT could restore the rhythmic circadian oscillation of clock genes, such as HvCCA1 and HvTOC1, in barley (*Hordeum vulgare*) seedlings, whose rhythmic phenotypes were disrupted due to environmental cold stress (5°C and 15°C). The results also confirmed that exogenous MT reduced the accumulation of key physiological indicators under cold stress, including MDA and soluble sugars (Chang et al., 2021).

Polyamines represent an important group of compounds known for their effects on enhancing cold tolerance in plants. A recent review by Shao et al. (2022) summarized the benefits of exogenous polyamine applications on plant stress responses to cold. Here, we briefly highlight a few recent studies. For instance, Gholizadeh et al. (2024) reported increases in morphophysiological and biochemical parameters in two wheat cultivars (‘Mihan’ and ‘Rakhshan’) grown under cold stress (4°C) when treated with putrescine and spermidine. The authors observed augmented activity of antioxidant-related enzymes such as catalase (CAT) and ascorbate peroxidase (APX), with concomitant upregulation of genes involved in polyamine synthesis (e.g., *TaADC*, *TaSAMDC*, and *TaSPDS*) and catabolism (*TaPAO11–7B* and *TaPAO11–7D*) (Gholizadeh et al., 2024). Similarly, He et al. (2024) demonstrated that exogenous spermidine enhanced cold resistance in 1-year-old apple branches by stabilizing tissue, reducing reactive oxygen species through increased proline content, and boosted antioxidant enzyme activity. They also noted the upregulation of key polyamine metabolism genes (MdADC1, MdSAMDC1, and MdSPDS1), alongside the cold-responsive genes (MdCBF1/2/3, MdCOR47, and MdKIN1). Collectively, these findings indicate that exogenous polyamines modulate metabolism and enhance antioxidant defenses, thereby improving cold tolerance in diverse crops.

## 8 Conclusions, gaps and perspectives

Rising environmental challenges, notably extreme temperatures, demand innovative solutions for crop production and protection. Low temperatures pose a significant threat, leading to substantial global agricultural losses. Plants respond to cold stress through various physiological mechanisms, including changes in gene expression, adjustments in cellular metabolism, and alterations in water and nutrient absorption. Therefore, finding effective solutions to address this issue is urgent.

Bioactive elicitors from macro- and microalgae enhance plant resilience to cold by activating natural defense mechanisms, promoting the expression of cold tolerance genes, and improving physiological processes. Commercial products derived from macroalgae, particularly *Ascophyllum nodosum*, are among the most widely used and show promise in boosting cold tolerance by increasing gene expression, reducing chlorophyll damage, and stimulating plant growth. Similarly, plant-derived biostimulants, such as extracts and soil microorganisms, can promote resilience in crops, by enhancing nutrient uptake, improving overall plant health, and stimulating natural defense mechanisms.

One of the key aspects highlighted in this review is the use of commercial products like Acadian^®^ and Algafect^®^, along with natural extracts, microorganisms and compounds such as glycine betaine and salicylic acid, in enhancing plant tolerance to low temperatures. These inputs have shown promising results in improving plant growth, root development, antioxidant activity, and gene expression related to cold stress.

However, several potential research gaps remain regarding broader applicability, long-term effects, optimal dosages, and practical implementation in agricultural settings. Further investigation in this area is essential to develop more effective strategies for mitigating cold stress in crop systems. This research could ultimately bridge the gap between laboratory findings and practical agricultural solutions for challenging climates.

Investigating the potential synergy between melatonin and other stress-responsive compounds or hormones, e.g., remains an interesting avenue. Combining melatonin with other biostimulants, such as algae-based products, may unlock enhanced stress tolerance that surpasses the capabilities of each compound when used in isolation.

There is still a need for improved guidelines and analysis parameters for determining whether a product qualifies as a biostimulant, whether it derives from a terrestrial or marine organism, or from purified molecules. Additionally, it is crucial to ascertain the most effective concentrations, optimal timing, and suitable delivery methods, taking into account variations among plant species and stress conditions. An essential aspect of research involves understanding the kinetics of absorption and distribution of certain molecules within plants.
